# Mitochondrial genome diversity of *Balamuthia mandrillaris* revealed by a fatal case of granulomatous amoebic encephalitis

**DOI:** 10.3389/fmicb.2023.1162963

**Published:** 2023-05-05

**Authors:** Cherie Tsz-Yiu Law, Thirapa Nivesvivat, Qing Xiong, Kasem Kulkeaw, Ling Shi, Pichet Ruenchit, Detchvijitr Suwanpakdee, Piradee Suwanpakdee, Nongnat Tongkrajang, Patsharaporn T. Sarasombath, Stephen Kwok-Wing Tsui

**Affiliations:** ^1^School of Biomedical Sciences, The Chinese University of Hong Kong, Shatin, Hong Kong SAR, China; ^2^Hong Kong Bioinformatics Centre, The Chinese University of Hong Kong, Shatin, Hong Kong SAR, China; ^3^Infectious Disease Unit, Department of Pediatrics, Phramongkutklao Hospital, Bangkok, Thailand; ^4^Siriraj Integrative Center for Neglected Parasitic Diseases, Department of Parasitology, Faculty of Medicine Siriraj Hospital, Mahidol University, Bangkok, Thailand; ^5^Neurology Division, Department of Pediatrics, Phramongkutklao Hospital, Bangkok, Thailand

**Keywords:** granulomatous amoebic encephalitis, *Balamuthia mandrillaris*, mitochondrial genome, free-living amoeba, ribosomal protein S3, neglected diseases, genotyping

## Abstract

**Introduction:**

*Balamuthia* (*B.*) *mandrillaris* is a free-living amoeba that can cause rare yet fatal granulomatous amoebic encephalitis (GAE). However, efficacious treatment for GAE is currently unavailable, especially when genomic studies on *B. mandrillaris* are limited.

**Methods:**

In this study, *B. mandrillaris* strain KM-20 was isolated from the brain tissue of a GAE patient, and its mitochondrial genome was *de novo* assembled using high-coverage Nanopore long reads and Illumina short reads.

**Results and Discussion:**

Phylogenetic and comparative analyses revealed a range of diversification in the mitochondrial genome of KM-20 and nine other *B. mandrillaris* strains. According to the mitochondrial genome alignment, one of the most variable regions was observed in the ribosomal protein S3 (*rps3*), which was caused by an array of novel protein tandem repeats. The repeating units in the *rps3* protein tandem region present significant copy number variations (CNVs) among *B. mandrillaris* strains and suggest KM-20 as the most divergent strain for its highly variable sequence and highest copy number in *rps3*. Moreover, mitochondrial heteroplasmy was observed in strain V039, and two genotypes of *rps3* are caused by the CNVs in the tandem repeats. Taken together, the copy number and sequence variations of the protein tandem repeats enable *rps3* to be a perfect target for clinical genotyping assay for *B. mandrillaris*. The mitochondrial genome diversity of *B. mandrillaris* paves the way to investigate the phylogeny and diversification of pathogenic amoebae.

## 1. Background

*Balamuthia mandrillaris* is one of the free-living amoeba species that can cause brain infections in humans besides *Acanthamoeba* spp., *Naegleria fowleri*, and *Sappinia* spp. (Visvesvara et al., 2007; Visvesvara, 2013, 2014). *B. mandrillaris* can enter the human body through the skin via wound or inhalation, and the infection causes a cutaneous lesion or brain infection called GAE, with up to 95% mortality rate (Matin et al., 2008; Siddiqui and Khan, 2008; Visvesvara, 2013, 2014; Vollmer and Glaser, 2016). This species was first identified from the brain of a pregnant mandrill baboon at the San Diego Wildlife Park in 1986, and the first human brain infection was reported in 1990 (Visvesvara et al., 1990). Since then, more than 200 cases of *Balamuthia* encephalitis have been reported worldwide (Intalapaporn et al., 2004; Cope et al., 2019; Wang et al., 2020). Due to the rarity and non-specific presentations of GAE, the diagnosis is usually delayed and often made postmortem (Schuster et al., 2009).

Unlike other free-living amoebae, which can be cultured on agar overlaid with bacteria, this organism needs to be grown on mammalian cell monolayer cultures such as monkey kidney cells, human lung fibroblasts, and human neuroblastoma cells (Schuster, 2002). Thus, the diagnosis of *B. mandrillaris* infection mainly relies on other laboratory methods including serology and genotyping based on the molecular marker in mitochondrial DNA (da Rocha-Azevedo et al., 2009). Currently, only two complete genomes of *B. mandrillaris* are available, both of which were obtained from patients in the United States (Detering et al., 2015; Greninger et al., 2015). The genome sizes of the two assemblies are 44.3 and 67.6 Mbp, respectively (Detering et al., 2015; Greninger et al., 2015). A total of nine mitochondrial genomes of *B. mandrillaris* are available, but none of them were isolated from Asia (Detering et al., 2015; Greninger et al., 2015). A previous study compared the mitochondrial genome of seven *B. mandrillaris* strains isolated from patients and the environment in the United States (Greninger et al., 2015). In addition to the various lengths of mitochondrial DNA sequences, the phylogenetic tree shows three distinct lineages (Greninger et al., 2015).

Mitochondrial genomes are increasingly used for phylogenetic and epidemiological analyses. In addition, several antiprotozoal drugs including pentamidine exert their functions by interfering with mitochondrial metabolism (de Souza et al., 2009). Mitochondrial genome analysis of *B. mandrillaris* may provide further insights into the diversity within species and shed light on the functions of mitochondrial genes, which could serve as potential drug targets.

Using both Nanopore long-read and Illumina short-read sequencing data, we *de novo* assembled the mitochondrial genome of the *B. mandrillaris* strain isolated from Asia named strain KM-20. Phylogenetic and comparative analyses of KM-20 and nine other strains were performed to investigate the mitochondrial genome diversity among strains. Notably, a previous study has reported the difference in the mitochondrial *rps3* gene, but the authors suggested that the difference is due to a putative intron or intergenic region (Greninger et al., 2015). In this study, our results demonstrated that the diversity of the *rps3* length is attributed to an array of protein tandem repeats, and the number of repeating units is different among *B. mandrillaris* strains.

## 2. Materials and methods

### 2.1. *Balamuthia mandrillaris* culture and DNA extraction

*Balamuthia mandrillaris* strain KM-20 was obtained by inoculating the left frontoparietal brain tissue of the GAE patient reported here in a monolayer of human lung carcinoma A549 cells in Dulbecco’s modified Eagle medium plus 10% fetal bovine serum at 37°C with 5% CO_2_, following the protocol as previously described (Schuster et al., 2009). The amoeba was first observed in the culture 4 weeks after inoculation and was maintained in a culture with A549 cells at 37°C with 5% CO_2_ (Schuster, 2002). *B. mandrillaris* strains V039 (50209) and V416 (PRA-290) were obtained from ATCC (Manassas, VA, United States) and maintained in culture media containing human neuroblastoma SH-SY5Y cells at 37°C with 5% CO_2_. DNA extractions of the amoeba were performed using a QIAmp DNA Mini Kit (Qiagen, Hilden, Germany), following the manufacturer’s protocol for isolating DNA from cell cultures.

### 2.2. DNA sequencing, assembly, and annotation

*Balamuthia mandrillaris* KM-20 genomic DNA was sequenced using Oxford Nanopore GridION Mk1 with a Ligation Sequencing Kit (SQK-LSK109) on an R9.4.1 MinION flow cell and an Illumina NovaSeq 6,000 sequencing system. The mitochondrial genome of *B. mandrillaris* strains 2046, V039, BeN, GAM-19, OK1, RP5, SAM, V188, and V451 (Table 1) was downloaded and used as a reference to map against the raw reads of KM-20 using Minimap2 (v2.20) to identify the mitochondrial DNA sequences of KM-20 (Li, 2018). The identified sequences were assembled into a single contig using Flye (v2.8.3) and subsequently polished by Illumina data using Pilon (v1.24) (Walker et al., 2014; Kolmogorov et al., 2019). The mitochondrial genome of KM-20 was visualized in Proksee (Stothard and Wishart, 2005; Grant and Stothard, 2008). The coding genes, introns, and novel open reading frames were identified by MITOS WebServer and GeSeq (v2.03) (Bernt et al., 2013; Tillich et al., 2017). The transfer RNA (tRNA) annotation was performed by GeSeq with ARAGORN (v1.2.38), and the rRNA subunit genes were checked by RNAweasel (Laslett and Canback, 2004; Lang et al., 2007). The coverage of the mitochondrial genome of *B. mandrillaris* KM-20 was obtained by mapping the nanopore raw reads to the assembled mitochondrial genome by Minimap2 (v2.20), and the mapping coverage was obtained using SAMtools (v1.5) (Li, 2018; Danecek et al., 2021). The *B*. *mandrillaris* KM-20 mitochondrial genome has been deposited in the National Center for Biotechnology Information (NCBI) under the accession number OM994889.

**Table 1:** *Balamuthia mandrillaris* strains used in this study.

Strain	Accession no.	Location	Source	Sequencing technology	References
KM-20	OM994889	Thailand	4-year-old girl, cultured on A549 feeder cells	Oxford Nanopore, Illumina	Current study
2046	KP888565	California	26-year-old man, survivor, cultured on Vero cells	Illumina	Vollmer and Glaser (2016) and Greninger et al. (2015)
CDC-V039	CM003363	California	3-year, 10-month-old pregnant mandrill from the San Diego Zoo Wild Animal Park, axenic culture	PacBio	Visvesvara et al. (1990) and Detering et al. (2015)
V039^a^	KT175741	California	3-year, 10-month-old pregnant mandrill from the San Diego Zoo Wild Animal Park, cultured on Vero cells	Illumina	Visvesvara et al. (1990) and Greninger et al. (2015)
V451	KT030670	New York	6-year-old girl, cultured on Vero cells	Illumina	Greninger et al. (2015)
GAM-19	KT175739	–	V188-frozen stock	Illumina	Greninger et al. (2015)
RP5	KT030672	California	Environmental sample, cultured on Vero cells	Illumina	Greninger et al. (2015) and Schuster et al. (2003)
SAM	KT030673	California	3-year-old girl, isolated from brain	Illumina	Greninger et al. (2015) and Bakardjiev et al. (2003)
V188	KT175738	Georgia	59-year-old man, isolated from brain/skin lesion, cultured on Vero cells	Illumina	Greninger et al. (2015); Gordon et al. (1992)
V416^a^	AF477015	Australia	10-year-old girl, isolated from brain	-	Booton et al. (2003)
OK1	KT030671	California	Environmental sample, cultured on Vero cells	Illumina	Dunnebacke et al. (2004)
BeN	NC_027736	–	–	Illumina	Alexander LG (Unpublished)

The accession number, location, and source of the strains used in this study are presented in the table.

a Obtained from ATCC.

### 2.3. Comparative mitochondrial genome analysis

Concatenated sequence data of cytochrome oxidase subunit 1 (*cox1*), cytochrome oxidase subunit 3 (*cox3*), cytochrome b (*cob*), ATP synthase F0 subunit 6 (*atp6*), ATP synthase subunit alpha (*atpa*), NADH dehydrogenase subunit 1 (*nad1*), NADH dehydrogenase subunit 2 (*nad2*), NADH dehydrogenase subunit 3 (*nad3*), NADH dehydrogenase subunit 4 (*nad4*), NADH dehydrogenase subunit 5 (*nad5*), NADH dehydrogenase subunit 6 (*nad6*), NADH dehydrogenase subunit 7 (*nad7*), and NADH dehydrogenase subunit 9 (*nad9*) of 10 strains of *B. mandrillaris* were aligned with MAFFT (v7.487) (Kuraku et al., 2013). The same set of genes in a concatenated sequence of *A. castellanii* (GenBank Accession number: U12386.1) was chosen as the outgroup. The alignment was imported into MEGA-X (v10.2.6) to perform phylogenetic analysis, and a maximum likelihood phylogenetic tree was computed using a JTT matrix-based model, with a bootstrap value of 1,000 (Jones et al., 1992; Kumar et al., 2018). The phylogenetic data were subsequently visualized using the Interactive Tree of Life (iTOL) (v5) (Letunic and Bork, 2021). The phylogenetic relationship and the mitochondrial sequences of 10 strains of *B. mandrillaris* were imported to AliTV for comparison and visualization (Ankenbrand et al., 2017). Regions with a low link identity were further aligned and examined by Clustal Omega (v1.2.4) (Sievers et al., 2011).

The *rps3* protein tandem repeat sequences of all *B. mandrillaris* strains were extracted to perform a phylogenetic analysis using MEGA-X (v10.2.6) (Kumar et al., 2018). A parent tree with all protein tandem repeat sequences was constructed by the maximum likelihood method and JTT matrix-based model with a bootstrap value of 1,000 (Jones et al., 1992; Kumar et al., 2018). A subtree of the protein tandem repeats was constructed by removing the most conserved branch of repeating units and the fourth repeating unit of KM-20 *rps3* from the parent tree. The remaining sequences were, then, used to construct a phylogenetic subtree by the maximum likelihood method and JTT matrix-based model and a bootstrap value of 1,000 (Jones et al., 1992). The phylogenetic data were visualized using iTOL (v5) (Letunic and Bork, 2021).

## 3. Results

### 3.1. *De novo* assembly and annotation

**Figure 1:**
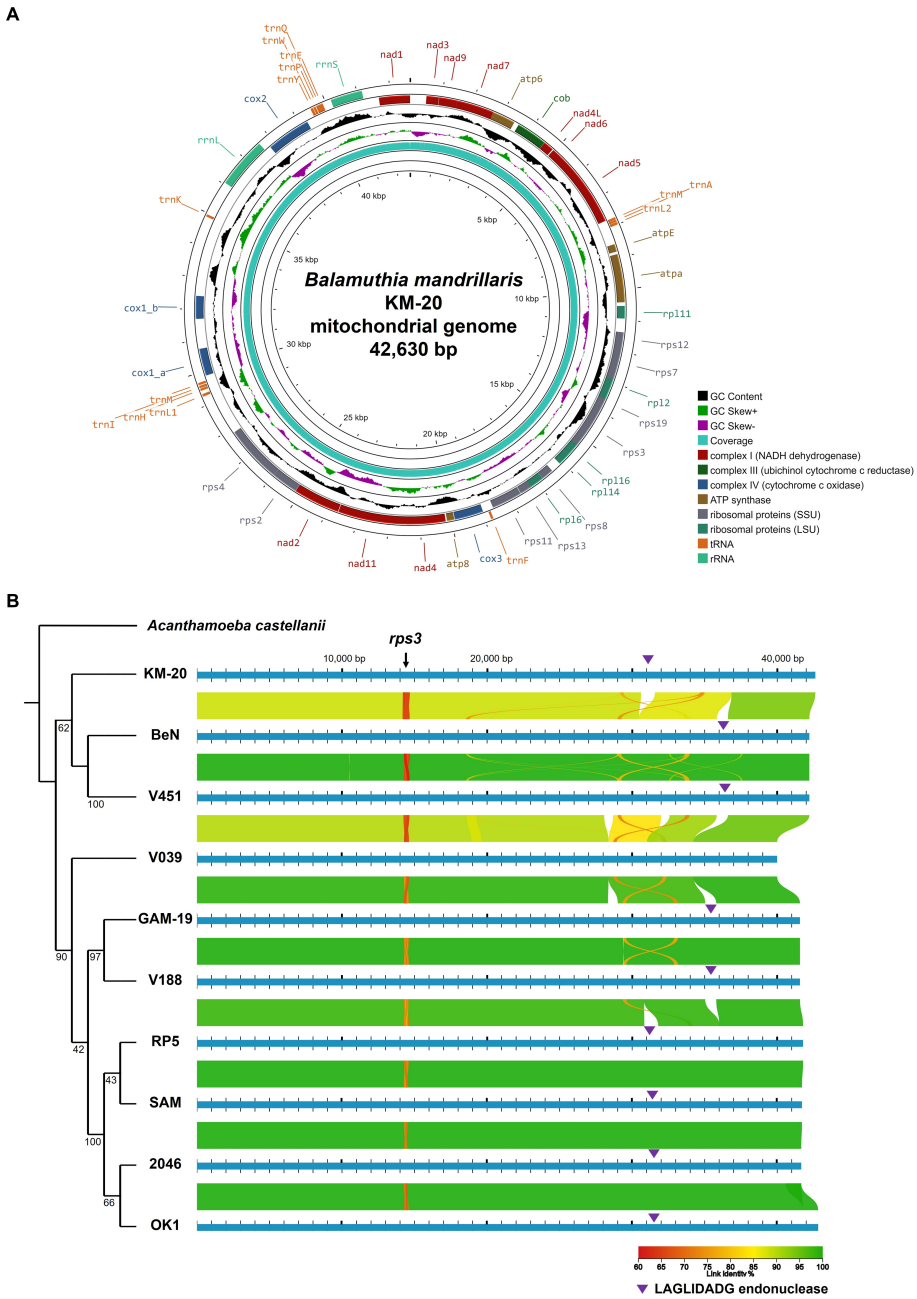
The mitochondrial genome of *B. mandrillaris* KM-20 and mitochondrial comparison among 10 strains of *B. mandrillaris.* **(A)** The whole mitochondrial genome of *B. mandrillaris* KM-20. The circular mitochondrial map depicts 33 protein-coding, two rRNA, and 13 tRNA genes. The average coverage of the mitochondrial genome assembly is 4464.09×, and the highest site coverage is 5,263×. The height of the innermost ring is calculated by dividing the site coverage by 5,263. **(B)** Phylogenetic relationship and mitochondrial genome alignment of 10 *B. mandrillaris* strains. Syntenic comparisons of linear mitochondrial chromosomal maps of 10 *B. mandrillaris* strains are visualized on AliTV software. Phylogenetic analysis was performed using concatenated sequences of *B. mandrillaris*, with *A. castellani* chosen as an outgroup. The phylogenetic relationship between 10 strains of *B. mandrillaris* is retained for mitochondrial genome comparison. The line color represents the percentage of linked sequence identity. A red-colored variable region with approximately 70% link identity was identified at approximately 14,500 bp in all strains of *B. mandrillaris,* which codes for the *rps3* gene. The purple triangle indicates the LAGLIDADG-endonuclease. Branch lengths are not drawn to proportion, and bootstrap values are shown for each node.

The brain debridement of the GAE patient was sent for culture on A549 cell lines. Genomic DNA of *B. mandrillaris* KM-20 was extracted from the culture and sequenced to obtain a total of 7.66 Gb data using Oxford Nanopore long-read sequencing technology and 23Gb data using the Illumina NovaSeq 6,000 system. A circular mitochondrial genome of the *B. mandrillaris* KM-20 was *de novo* assembled in size of 42,630 bp and 35.34% GC content (Figure 1A). A total of 33 protein-coding, two rRNA, and 13 tRNA genes were annotated and located in the plus strand of the mitochondrial genome of *B. mandrillaris* KM-20 (Figure 1A). The protein-coding genes were classified into five groups, namely, ribosomal protein, NADH dehydrogenase, ATP synthase, cytochrome c oxidase, and cytochrome b.

According to a previous report, the mitochondrial genome size of *B. mandrillaris* strains ranges from 39,996 bp to 42,823 bp (Supplementary Table S2) (Greninger et al., 2015). The mitochondrial *cox1* gene of KM-20 is interrupted by a LAGLIDADG endonuclease-containing group IB intron (Supplementary Figure S2), which was also observed in four other strains, namely, 2046, SAM, RP5, and OK1 (Greninger et al., 2015). The length of LAGLIDADG endonuclease in these five strains ranges from 281 to 283 amino acids. For other strains, including, V451, V188, BeN, and GAM-19, the LAGLIDADG endonuclease is in the 23S rRNA gene, instead of the *cox1* gene (Greninger et al., 2015). V039 is the only strain that has no LAGLIDADG endonuclease inserted in protein-coding genes (Greninger et al., 2015).

### 3.2. Comparative analysis of *Balamuthia mandrillaris* strains

The phylogenetic analysis of *B. mandrillaris* mitochondrial genomes divided 10 strains into two clades and suggested KM-20 as the most distant strain (Figure 1B). In agreement with a previous study (Greninger et al., 2015), the four California strains (RP5, SAM, 2046, and OK1) formed a highly conserved cluster in the phylogenetic tree.

To perform a global mitochondrial genes comparison, the coding genes of 10 *B. mandrillaris* strains were compared in a matrix of pairwise identity percentage using KM-20 as a reference, and *rps3* was the only mitochondrial gene that has a percentage identity lower than 85% (Supplementary Figure S3). To further investigate the genomic diversity of *B. mandrillaris*, the mitochondrial genomic architectures of 10 strains were visualized by AliTV, and the result revealed a generally conserved gene synteny (Figure 1B) (Stajich et al., 2002; Harris, 2007; Ankenbrand et al., 2017). A break in synteny indicated in the purple triangle was observed in *cox1* and 23S rRNA, which corresponds to the introns that contain LAGLIDADG endonuclease. Other mapping gaps were caused by sequences missing in strain V039 and are not intron after manual checking. A region with only 70% link identity at approximately 14,500 bp was identified in the mitochondrial genome, and the variable position was confirmed to be the *rps3* gene. Interestingly, multiple sequence alignment of rps3 protein sequences of 10 *B. mandrillaris* strains revealed that the variation could be attributed to an array of protein tandem repeats (Supplementary Figure S4). The tandem repeat unit in *rps3* is named the R unit, and each R unit is composed of 17 amino acid residues, most of which started with four consensus amino acids, namely, arginine (R), proline (P), tryptophan (W), and leucine (L) (Supplementary Figure S4). KM-20 has seven R units, which is the highest number among all strains; V451 and BeN have six; GAM-19, V188, V039, RP5, and OK1 have five; SAM and 2046 have four and three R units, respectively. Despite the amino acid sequence within each R unit being conserved, the nucleotide sequences are highly degenerated and can be differentiated from each other. The identified repeats, therefore, are not due to sequencing error or collapse (Supplementary Figure S5). The length of the protein tandem repeat region in rps3 ranges from 51 to 121 amino acid residues. The CNVs of the R units account for the difference in *rps3* length, which makes *rps3* a promising target for strain identification and genotyping of *B. mandrillaris* (Greninger et al., 2015).

**Figure 2:**
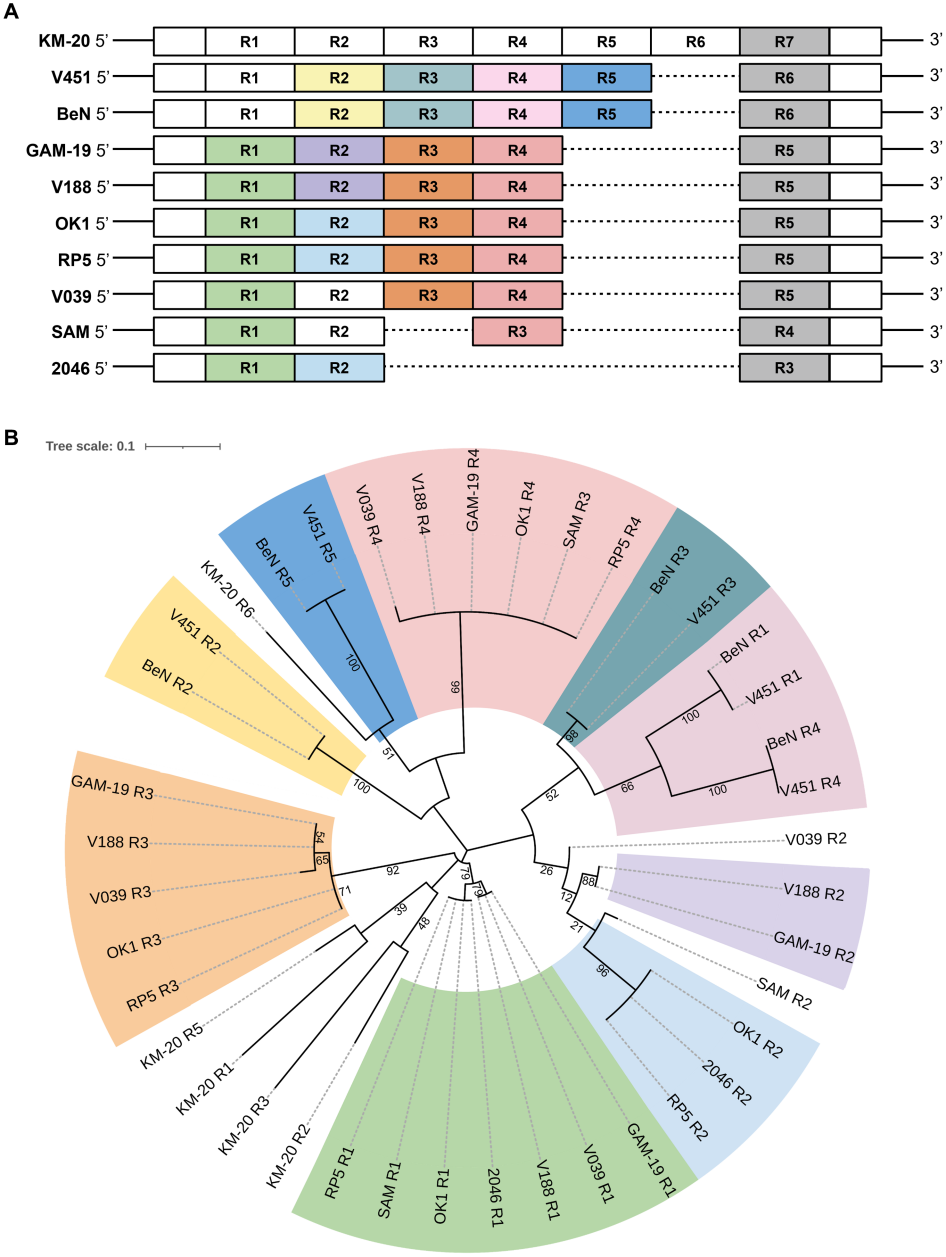
Distribution of the protein tandem repeats in rps3 with their phylogenetic relationship matching the colors. **(A)** Distribution of protein tandem repeats in rps3. Sequence segments are not drawn to scale. KM-20 has seven R units; V451 and BeN have six; GAM-19, V188, V039, RP5, and OK1 strain have five; SAM strain has four, and the 2046 strain has three R units in *rps3*. **(B)** Phylogenetic analysis of rps3 R units. The subtree of rps3 R units shows nine main clades. R units that are not phylogenetically clustered with other repeating units are shaded in white, such as all R units of KM-20, R2 of SAM, and R2 of V039.

The distribution of R units in *rps3* was illustrated with colors according to their phylogenetic relationship (Figure 2). A parent phylogenetic tree was constructed with all R units in 10 *B. mandrillaris* strains (Supplementary Figure S6). The last R units of all strains form a highly conserved branch in the parent phylogenetic tree and are colored in gray (Supplementary Figure S6). To better explore the R unit divergence, the gray-colored R units together with the most distant R4 unit of KM-20 were removed to construct a high-quality phylogenetic subtree (Figure 2B). The R units in 10 *B. mandrillaris* strains can be divided into nine main clades (Figure 2B).

**Figure 3:**
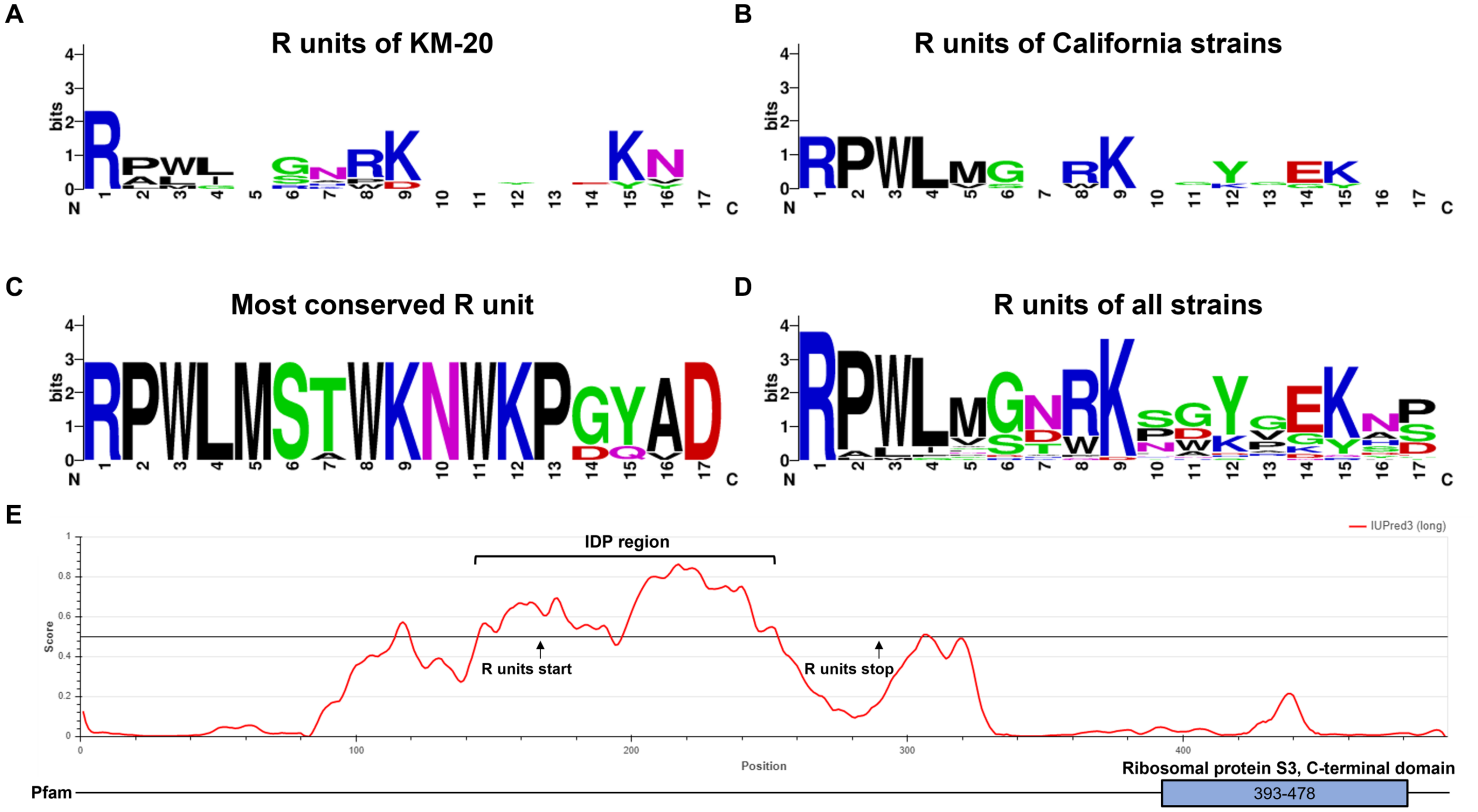
Tandem repeat consensus amino acid sequence of *B. mandrillaris* rps3. **(A)** Tandem repeat consensus amino acid sequence of KM-20 rps3. **(B)** Tandem repeat consensus amino acid sequence of California strains (RP5, 2046, SAM, and OK1 strain) rps3. **(C)** Tandem repeat consensus amino acid sequence of the most conserved R units, which are the sequences nearest to the C-terminal of rps3. **(D)** Tandem repeat consensus amino acid sequence of R units in 10 *B. mandrillaris* strains. The consensus sequence for each repeat is RPWL-G-RK--Y-EK--. The WebLogo consists of stacks of letters as follows: one stack for each position in the sequence. The overall height of the stack shows the sequence conservation at that position, which is measured in bits, while the height of the symbols within the stack represents the relative frequency of each amino acid at that position. Amino acids are colored according to their chemical properties as follows: polar amino acids are colored in green; basic are in blue; acidic are in red, and hydrophobic amino acids are in black. **(E)** Intrinsically disordered protein region was identified in the tandemly repeated region of KM-20 rps3. The functional ribosomal protein S3 is identified in the C-terminal domain.

A consensus sequence motif was generated to identify the conserved amino acid residues in different clusters by WebLogo (Figure 3) (Schneider and Stephens, 1990; Crooks et al., 2004). The R units of KM-20 are highly variable (Figure 3A), while the R units of the California strains (RP5, SAM, 2046, and OK1) are significantly conserved and start with RPWL amino acid residues (Figure 3B). The consensus sequences of the gray-colored R units in Figure 2A and overall R units from all strains are RPWLMSTWKNWKPGYAD and RPWL-G-RK--Y-EK--, respectively (Figures 3C,D). Positions 2–5 are populated by hydrophobic amino acids, which are colored in black (Figures 3A–D). The R units of all strains start with RPWL except R2, R3, and R4 of KM-20 and R5 of V451 and BeN, but the substituting amino acid residues, such as alanine, isoleucine, phenylalanine, and methionine, are also hydrophobic in nature, suggesting an N-terminal hydrophobic region is important for R units.

The general structure of rps3 was predicted to have four transmembrane helices and three cytosolic domains by InterPro88.0 (Jones et al., 2014; Blum et al., 2021) (Supplementary Figure S7). The R units and the C-terminal domain of rps3 are predicted to be in the cytosolic compartments (Supplementary Figure S7). The protein tandem repeats in rps3 were identified as an intrinsically disordered region (IDR) by IUPred3 (Figure 3E) (Erdős et al., 2021). IDRs have no well-defined three-dimensional structures but are dynamically disordered and can fluctuate rapidly through different conformations (Wright and Dyson, 2015). The disordered region is in position 145–253 and overlaps with the repeating R units of KM-20, which are in position 167–293. Further structural and molecular analysis may assist in understanding the function of this highly variable region of rps3.

## 4. Discussion

*Balamuthia mandrillaris* is one of the free-living amoebae that occasionally cause GAE in humans and animals (Visvesvara et al., 2007; Visvesvara, 2013; Visvesvara, 2014), which is often life-threatening with limited treatment options (Cope et al., 2019). Owing to the restricted number of documented cases worldwide, establishing a conclusive link between genotype variation and clinical manifestation poses a challenge. It is speculated that variations in the genotypes of *B. mandrillaris* may account for the dissimilar clinical presentations of its infection across diverse regions of the world. Retrospective reports from China and Peru demonstrated that the main clinical manifestations of *B. mandrillaris* infection are cutaneous lesions, which precede neurological involvement that develops several years later. In contrast, reported cases from the US presented solely with neurological symptoms, without any preceding skin lesion (Bravo and Seas, 2012; Wang et al., 2020), which is similar to the clinical presentation of our current case. Thus, the dissimilarity in disease aggressiveness and clinical manifestations could potentially stem in part from the genetic variability within the species. Unfortunately, mitochondrial genome sequences of the cases reported in Peru and China, apart from those in the US, are unavailable for comparison with the current case. To comprehend the genetic variation that could be associated with clinical manifestations, the mitochondrial genome of *B. mandrillaris* strain KM-20 was *de novo* assembled and annotated in this study. To our knowledge, this study reports the first complete mitochondrial genome of *B. mandrillaris* clinical isolate obtained from Asia, as others were sequenced from samples isolated in the US. By comparing the mitochondria of KM-20 with other strains collected from the non-Asian area, we found the mitochondrial genome diversity can be attributed to the LAGALIDADG-containing intron in either *cox1* or 23S rRNA and a novel array of protein tandem repeats in *rps3*, which raises questions about the functional roles of this region within mitochondria and cells. In addition to clinical genotyping, the CNVs and domain architecture of the *rps3* tandem repeat can also infer the phylogenetic relationship among strains. The close phylogenetic relationship of the last R units among strains suggested that they could be the most ancient R unit (Supplementary Figure S6). *Cox1* gene has been widely used for species identification, phylogeography, and phylogenetic inference studies; however, the efficacy of using other mitochondrial genes has become less explored (Luo et al., 2011). The variable region in *rps3* can provide additional information on the phylogenetic relationship among strains, such as the copy number and divergence of the R units in *rps3*.

The mitochondrial features of *B. mandrillaris* among strains are generally conserved in terms of gene synteny and coding sequence, except for the presence of LAGLIDADG-containing endonuclease in either *cox1* or 23S rRNA, and the CNVs of protein tandem repeats in *rps3*. LAGLIDADG is a homing endonuclease occasionally included in the Group I self-splicing introns, which can cleave an intronless allele, resulting in the insertion of an intron and endonuclease into the previous intronless allele (Heidel and Glöckner, 2008). Group I introns are commonly found in fungi and protist nuclear rRNA genes as well as in organellar genomes, yet other organisms usually have no Group I introns in the genome (Haugen et al., 2007). Electrophoretic mobility shift assay and DNA cleavage assays can be further performed to identify the target sites of the LAGLIDADG-containing endonuclease (Grindl et al., 1998).

It is known that *rps3* plays a critical role in ribosome biogenesis and DNA repair in humans (Kim et al., 2013). Under stress conditions that promote DNA damage in which the cellular reactive oxygen species level increase, *rps3* accumulates in the mitochondria to repair damaged DNA (Kim et al., 2013). The analysis of ribosomal protein genes is currently lacking since metazoan mitochondrial genomes do not carry ribosomal protein genes (Gray et al., 1998; Heidel and Glöckner, 2008). The function of the *rps3* gene and the implication of CNV in the *rps3* tandem region in *B. mandrillaris* are currently unknown. Alterations in ribosomal genes in other species are shown to be related to adaptation and survival (Finken et al., 1993; Chittum and Champney, 1994). For example, amino acid changes in *rps12* in *Mycobacterium tuberculosis* are adaptive for streptomycin resistance (Finken et al., 1993). Mutation in the ribosomal protein gene sequence in *Escherichia coli* is related to erythromycin resistance (Chittum and Champney, 1994). The function of CNVs in *rps3* tandem repeats will be interesting to further investigate.

The rps3 protein sequence of KM-20 was searched in the NCBI non-redundant (NR) database using BLASTp on 26 September 2022. The result showed a significant match to the C-terminal domain of rps3 with an e-value of 1.88e-05 (Supplementary Figure S8a), confirming the gene annotation. The percentage identities of the rps3 protein sequence of KM-20 to humans, *Saccharomyces cerevisiae*, *Dictyostelium discoideum*, and *A. castellanii* were calculated by Clustal Omega (v1.2.4), which are 19.69%, 17.28%, 18.37%, and 30.53%, respectively. The C-terminal region of rps3 of all *B. mandrillaris* strains constituted 113 amino acid residues and shared pairwise identities in the range of 99.12% to 100%, suggesting high conservation of rps3 C-terminal within the species. In contrast, high variation was observed in the rps3 sequence between *B. mandrillaris* and other species, including *A. castellanii*. Surprisingly, the rps3 of *B. mandrillaris* is more similar to bacteria than to other amoebae, as the top BLASTp matches of KM-20 rps3 are sequences of bacteria such as *Candidatus Calescamantes*, *Caldiserica* bacteria, and *Metallibacterium scheffleri* (e-value: 4e-06, 5e-06, and 7e-06, respectively) (Supplementary Figure S8b). However, the rps3 protein sequence of humans, mice, *Drosophila melanogaster*, *S. cerevisiae*, and *D. discoideum* is relatively conserved, with pairwise amino acid residues identities in the range of 59.15% to 99.59%. It is speculated that the origin of the *rps3* gene in *B. mandrillaris* is different from other amoeba species. Further investigations can be performed to investigate the evolutionary origin of *rps3* in amoeba species.

Amoebae inhabit a wide range of ecological niches and rapid adaptation to new environments is advantageous to their survival. Varying the number of tandemly arrayed repeating units can increase the genomic sequence diversity and may enable the organisms to adapt to new environments relatively quicker and undergo more rapid and error-prone evolution than non-repeat-containing proteins (Marcotte et al., 1999; Jernigan and Bordenstein, 2015). All 10 strains of *B. mandrillaris* contain an array of protein tandem repeats in the *rps3* gene, which is not found in other amoebae, including *A. castellani* and *N. fowleri*, raising questions about the function of the protein tandem repeats in *B. mandrillaris* exclusively. Although unicellular organisms can have significant deviations from typical animal mitochondrial genomes (Lavrov and Pett, 2016), a CNV in the mitochondrial coding region with substantial size variation among strains has not been reported in amoeba before. The conserved specific residues within each R unit may be critical for the structure or function, despite the precise number of repeats and the amino acid sequence may vary among strains (Javadi and Itzhaki, 2013).

A total of two genotypes of *rps3* were observed in strain V039, and the genetic difference lies in a 102 bp insertion in *rps3* which accounts for two extra R units (Detering et al., 2015; Greninger et al., 2015) (Supplementary Figure S9a). Both samples of V039 were isolated from the brain of a pregnant baboon that died from meningoencephalitis at the San Diego Zoo Wild Animal Park in 1990 but was subsequently cultured in different culture media (Detering et al., 2015; Greninger et al., 2015). The mitochondrial genome of the axenic cultivated CDC-V039 has a size of 39,894 bp while the other published mitochondrial genome of V039 was cultured on Vero cells and has a size of 39,996 bp (Detering et al., 2015; Greninger et al., 2015) (Supplementary Figure S9b). Multiple sequence alignment has confirmed that the axenic CDC-V039 has three R units, whereas the Vero cell-cultured V039 has five, suggesting the possibility of mitochondrial heteroplasmy in *B. mandrillaris*. To verify whether the mitochondrial heteroplasmy of *B. mandrillaris* can be observed under certain conditions, we examined whether the change in culture media or temperature would induce mitochondrial heteroplasmy. However, we did not observe any evidence of mitochondrial heteroplasmy in the *rps3* gene of three *B. mandrillaris* strains including KM-20, V039, and V416 under various culture temperatures and culture medium conditions (Supplementary Figure S10).

## 5. Conclusion

In this study, we *de novo* assembled and annotated the complete mitochondrial genome of *B. mandrillaris* KM-20 using long-read and short-read sequencing data. Our comparative results explored the mitochondrial genome diversity among *B. mandrillaris* strains and revealed that one of the mitochondrial variations arises from an array of protein tandem repeats in the *rps3* gene, which has not been reported in other amoebae before. The copy number and sequence variations of the protein tandem repeats enable *rps3* to be a promising gene target for genotyping *B. mandrillaris* and can provide additional phylogenetic information. Collectively, this comparative mitochondrial genome analysis paves the way to investigate the evolution and genetic diversity of *B. mandrillaris* and other pathogenic amoebae.

## Data availability statement

The datasets presented in this study can be found in online repositories. The names of the repository/repositories and accession number(s) can be found in the article/Supplementary material.

## Ethics statement

The studies involving human participants were reviewed and approved by Siriraj Institutional Review Board of research involving human subjects (SIRB) (COA no. Si 806/2020). Risk Management Taskforce, Mahidol University, Thailand with approval no. SI2020-035. Written informed consent to participate in this study was provided by the participants’ legal guardian/next of kin. Written informed consent was obtained from the minor(s)’ legal guardian/next of kin for the publication of any potentially identifiable images or data included in this article.

## Author contributions

CL, TN, QX, KK, and PTS contributed to the conception and design of the study. TN, DS, and PS provided clinical data. CL, PTS, KK, LS, PR, and NT were responsible for the laboratory study. CL, TN, and PTS wrote the first draft of the manuscript. CL, QX, PTS, KK, and ST finalized the manuscript. All authors contributed to the article and approved the submitted version.

## Funding

This study was supported by grants from the General Research Fund from Research Grants Council (Reference numbers: 464710, 475113, 14119219, 14119420, and 14175617), Health and Medical Research Fund from Food and Health Bureau (Reference numbers: 06171016 and 07181266), and Theme-based Research Scheme project from the Research Grants Council (Reference number: T11-712/19 N) in Hong Kong. This study was also supported by a grant from the Faculty of Medicine Siriraj Hospital, Mahidol University (Grant number R016433002) and was carried out under the Siriraj Integrative Center for Neglected Parasitic Diseases, Faculty of Medicine Siriraj Hospital, Mahidol University. KK, PR, and PTS also received Chalermphrakiat grants from the Faculty of Medicine Siriraj Hospital, Mahidol University.

## Conflict of interest

The authors declare that the research was conducted in the absence of any commercial or financial relationships that could be construed as a potential conflict of interest.

## Publisher’s note

All claims expressed in this article are solely those of the authors and do not necessarily represent those of their affiliated organizations, or those of the publisher, the editors and the reviewers. Any product that may be evaluated in this article, or claim that may be made by its manufacturer, is not guaranteed or endorsed by the publisher.
