# Supplementary material for: Mitochondrial genome diversity of Balamuthia mandrillaris revealed by a fatal case of granulomatous amoebic encephalitis
Source: Front Microbiol. 2023 May 5;14:1162963. doi: 10.3389/fmicb.2023.1162963 (PMC10196457; doi:10.3389/fmicb.2023.1162963)
Supplement: Supplementary file 1 [file Data_Sheet_1.docx]

Supplementary Material

**Supplementary note: Details of clinical data and treatment outcome of the GAE case**

A 4-year-old Thai girl presented to the hospital with a 3-week history of intermittent headache in the forehead area with projectile vomiting. She had intense pain, especially at night causing sleep disturbance. Besides having this problem, she had good consciousness with no fever or neurological deficits. She had no significant exposure history including soil exposure, nasal aspiration during swimming in a freshwater reservoir, no chronic wound, or chronic sinusitis. She had no travel history outside the country. Her initial neurological examination was unremarkable except for having motor power grade IV in the right upper and lower extremities. Ophthalmologic examination revealed a normal eye exam, no papilledema, and no retinal hemorrhage. Her complete blood count examination revealed a normal hematocrit at 37.2 % and a normal white blood cell count at 5,700 /mm^3^ (neutrophils 65%, lymphocytes 32%, and monocytes 2%). A cerebrospinal fluid (CSF) examination was not initially performed. Brain magnetic resonance imaging (MRI) was performed and revealed two heterogeneous enhancing lesions. (Supplementary Fig. S1.1a and S1.1b). A brain biopsy at the frontoparietal lesion revealed an inflamed and necrotizing brain with a mixed infiltrate of inflammatory cells, mostly lymphocytes, and histiocytes. Brain biopsy of the lesion revealed a few large amphophilic oval-shaped structures with a single nucleus containing a halo around the karyosome, highly suggestive of amoebic trophozoites (Supplementary Fig. S1.1c). Due to the inability to differentiate the causative species of free-living amoebae in histological brain sections based on their morphology, additional investigations, including PCR and tissue culture, were conducted to verify the causative species. The brain tissue was subjected to molecular characterization of possible causative species including *B. mandrillaris*, *Acanthamoeba* spp., and *N. fowleri*, and the causative species was identified as *B. mandrillaris* by PCR-targeted 16S rRNA. Sanger DNA sequencing was performed for genes including 16S rRNA, *cox1*, and 18S rRNA of the causative *B. mandrillaris* strain. The sequences of 16S rRNA and *cox1* shared 98.9% and 93.2% identities with those of *B. mandrillaris* strain SAM (GenBank accession: KT030673.1), respectively. The 18S rRNA sequence shared 99.5% identity with that of *B. mandrillaris* isolate Itson-1 (GenBank accession: KF874819.1). The sequencing results confirmed the diagnosis of GAE and the causative species was suggested to be a new *B. mandrillaris* strain named KM-20. This is the third confirmed case of *B. mandrillaris* infection in Thailand and the second case without a cutaneous lesion (Intalapaporn et al., 2004; Krasaelap et al., 2013).

Brain MRI after the biopsy showed an increased size of both lesions (Supplementary Fig. S1.2a and Fig. S1.2b). The peripheral part of the larger lesion showed contiguous rim-enhancing representing necrotic portions in the abscesses (Supplementary Fig. S1.2a). The smaller lesion developed into a cluster of rim-enhancing lesions (Supplementary Fig. S1.2b). New multiple lesions at the right temporo-occipital area were detected, compatible with the progression of multifocal brain abscesses (Supplementary Fig. S1.2c). Physical examination showed motor power grade IV in the right upper and lower extremities. The patient received a combination therapy of fluconazole (12 mg/kg/day), co-trimoxazole (20 mg/kg/day), rifampicin (15 mg/kg/day), and dexamethasone (0.35 mg/kg/day) for 1 month. One month later, she developed a high-grade fever with right arm weakness and progressive headache while still on continuous combination therapy with fluconazole, co-trimoxazole, rifampicin, and prednisolone. Physical examination revealed high-grade fever at 38.5^◦^C with right-sided weakness, motor power grade II at both upper and lower extremities with hyperreflexia, and persistent clonus. A follow-up brain MRI revealed a significant progression of the lesions. Multiple brain abscesses in bilateral cerebral hemispheres were observed with abnormal leptomeningeal enhancement at the perimesencephalic cistern and bilateral sylvian cisterns, along with the courses of bilateral middle cerebral arteries (Supplementary Fig. S1.2d and Fig. S1.2e). The patient then received craniotomy and partial abscess resection to release intracranial pressure and the tissue debridement was sent for culture on A549 cell lines. The culture was positive with the presence of *B. mandrillaris* trophozoites at 4 weeks after inoculation (Supplementary Fig. S1.1d). Intraoperative findings revealed moderate brain edema and non-suppurative slough necrotic tissue on the left frontal and parietal lobes (Supplementary Fig. S1f). The patient received a combination therapy of fluconazole (12 mg/kg/day), co-trimoxazole (20 mg/kg/day), rifampicin (15 mg/kg/day), and dexamethasone (0.35 mg/kg/day). Six weeks later her condition deteriorates with a high-grade fever, and progressive right-sided weakness (motor power grade II in both lower and upper extremities) with hyperreflexia and persistent clonus. Craniotomy and partial abscess resection were performed to decrease intracranial pressure. The patient later developed intracranial hypertension, cardiac arrest, and died two weeks after the operation.

**Supplementary Table 1. PCR primers and cycling conditions used in this study.**

| Species / target | Primers | PCR cycling condition | Citation |
| --- | --- | --- | --- |
| *B. mandrillaris* / 16S rRNA | Balspec16S-Fwd:  5’-CGCATGTATGAAGAAGACCA-3’  Balspec16S-Rev:  5’-TTACCTATATAATTGTCGATACCA-3′ | 94^◦^C-5 min; 40 cycles of 94^◦^C-1 min, 54^◦^C-2 min, 72^◦^C-3 min; 72^◦^C-10 min, 4^◦^C-forever | Booton GC et al., 2003 |
| *B. mandrillaris* / 18S rRNA | Balcon18S-Fwd:  5’-CGTAACGGGTAACGGAGAATTAG-3’  Balcon18S-Rev:  5’-CCACCACCCATAGAATCAAGAA-3’ | 95^◦^C-5 min; 35 cycles of 95^◦^C-30 s, 51^◦^C-30 s, 72^◦^C-1 min; 72^◦^C-10 min, 4^◦^C-forever | Current study |
| *B. mandrillaris* / *cox1* | BalconCOX1-Fwd:  5’-CGAATCCTTCCCGTGTCTTT-3’  BalconCOI-Rev:  5’-TCCCAGCATACAGTCCATTTC-3’ | 95^◦^C-5 min; 35 cycles of 95^◦^C-30 s, 52^◦^C-30 s, 72^◦^C-1 min; 72^◦^C-10 min, 4^◦^C-forever | Current study |
| *B. mandrillaris* / *rps3* | HK-IPD1-Fwd:  5’-TTTATTCGTCGACYGTGGCT-3’  HK-IPD-Rev:  5’-CACAAACCAACGRAAAAACCC-3’ | 95^◦^C-5 min; 35 cycles of 95^◦^C-30 s, 61^◦^C-30 s, 72^◦^C-1 min; 72^◦^C-5 min, 4^◦^C-forever | Current study |
| *N. fowleri* / *ITS1* | Nf ITS1-Fwd:  5′-GAACCTGCGTAGGGATCATTT-3′  Nf ITS1-Rev:  5′-TTTCTTTTCCTCCCCTTATTA-3′ | 94^◦^C-5 min; 35 cycles of 94^◦^C-30 s, 55^◦^C-30 s, 72^◦^C-45 s; 72^◦^C-5 min, 4^◦^C-forever | Pélandakis M et al., 2000 |
| *Acanthamoeba spp*. / 18S rRNA | JDP1-Fwd:  5’-GGCCCAGATCGTTTACCGTGAA-3’  JDP2-Rev:  5’-TCTCACAAGCTGCTAGGGAGTCA-3’ | 95^◦^C-7 min; 35 cycles of 95^◦^C-1 min, 59^◦^C-30 s, 72^◦^C-1 min; 72^◦^C-1 min, 4^◦^C-forever | Schroeder JM et al., 2001 |

**
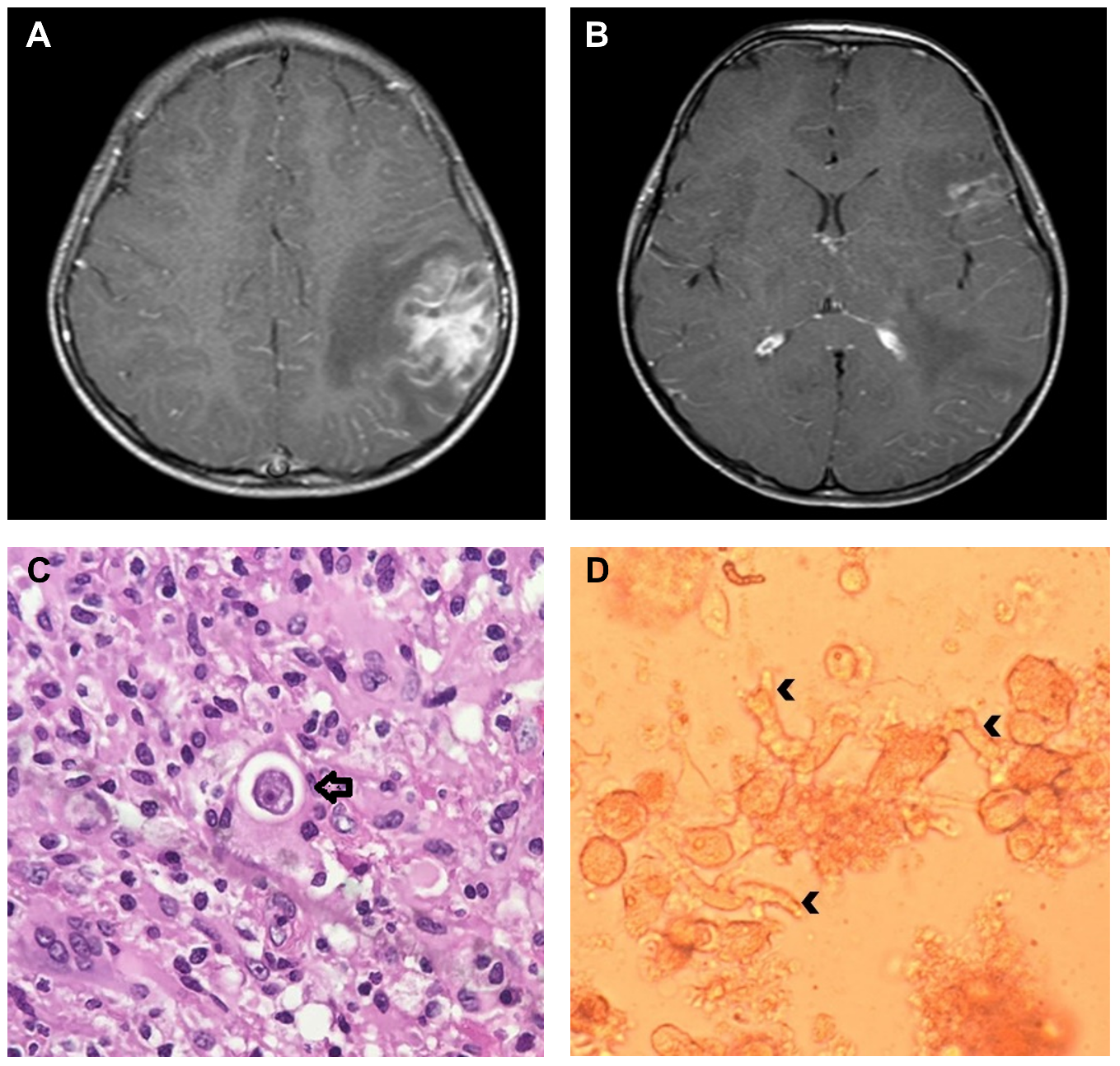
**

**Supplementary Fig S1.1. Brain imaging and laboratory findings from the reported case.** **(A, B)** The contrast-enhanced T1-weighted MRI brain reveals two heterogeneous enhancing lesions. **(A)** The larger lesion involved the left frontoparietal lobe showing a solid enhancing portion with multiple peripheral rim-enhancing lesions. **(B)** The smaller lesion at the left frontal lobe showed a partial rim-enhancing lesion. These two lesions involved both gray and white matter and extended to the brain surface with brain edema. **(C)** H&E staining of the patient’s brain tissue revealed an amoeba trophozoite with a single nucleus containing a large central karyosome surrounded by halo (arrow). The brain tissues contained necrotic tissue and inflammatory cell infiltration mostly lymphocytes and histiocytes were observed. **(D)** *B. mandrillaris* trophozoites were observed when co-cultured with A549 cell lines at 4 weeks post-inoculation. The trophozoites were pleomorphic with some showing broad, fragmented pseudopods (arrowhead).


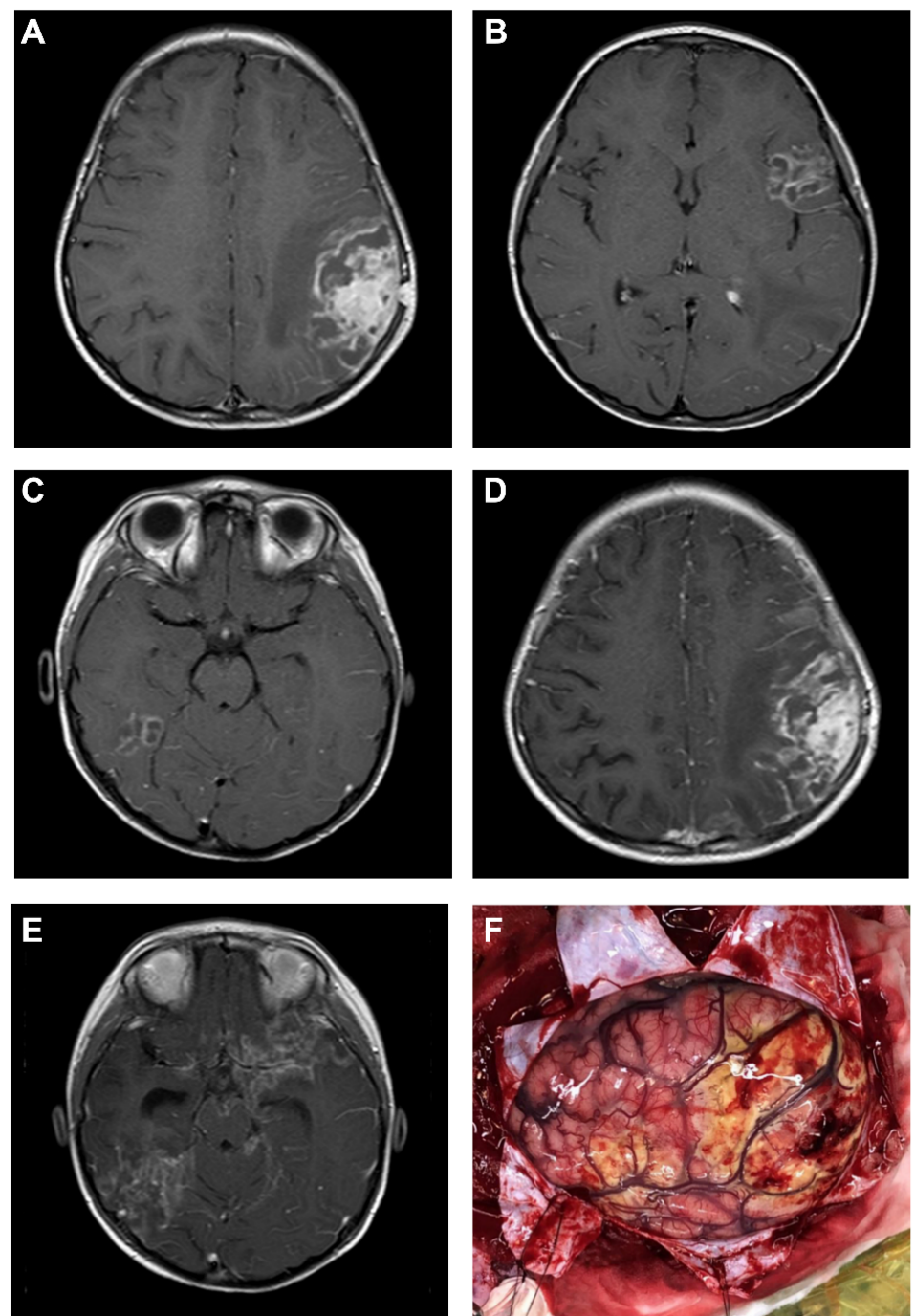


**Supplementary Fig. S1.2. Follow-up brain imaging showed significant progression of the lesion. (A-C)** The follow-up contrast-enhanced T1-weighted MRI brain after the biopsy showed an increase in the size of both lesions at the left frontoparietal lobe and left frontal lobe. **(A)** The peripheral part of the larger lesion at the left frontoparietal lobe showed contiguous rim-enhancing representing necrotic portions in the abscesses. **(B)** The smaller lesion at the left frontal lobe developed into a cluster of multiple rim-enhancing lesions. The perilesional brain edema progressed. **(C)** The other group of multiple rim-enhancing lesions at the right temporo-occipital area were newly detected. These findings are compatible with the progression of multifocal brain abscesses. **(D-E)** The follow-up contrast-enhanced T1-weighted MRI brain 2 months after treatment showed an increase in size and number of multiple brain abscesses in bilateral cerebral hemispheres. Abnormal enhancement of the leptomeninges at the perimesencephlic cistern and bilateral sylvian cisterns, along the courses of bilateral middle cerebral arteries was compatible with leptomeningeal involvement by the infection. **(F)** Intraoperative findings during tissue debridement revealed moderate brain edema and non-suppurative slough necrotic tissue on the left frontal and parietal lobes.

|  | KM-20 | | BeN | | V451 | | GAM-19 | | OK1 | | RP5 | | SAM | | V188 | | V039 | | 2046 | | |  |
| --- | --- | --- | --- | --- | --- | --- | --- | --- | --- | --- | --- | --- | --- | --- | --- | --- | --- | --- | --- | --- | --- | --- |
| Mitochondrial genome size (bp) | | 42,630 | | 42,217 | | 42,217 | | 41,570 | | 42,823 | | 41,784 | | 41,707 | | 41,571 | | 39,996 | | 41,656 | | |
| Number of protein-coding genes | | | | | | | | | | | | | | | | | | | | |  |  |
| NADH dehydrogenase | | 10 | | 10 | | 10 | | 10 | | 10 | | 10 | | 10 | | 10 | | 10 | | 10 | | |
| ATP synthase | | 4 | | 4 | | 4 | | 4 | | 4 | | 4 | | 4 | | 4 | | 4 | | 4 | | |
| Ribosomal protein | | 14 | | 14 | | 14 | | 14 | | 14 | | 14 | | 14 | | 14 | | 14 | | 13 | | |
| Cytochrome c oxidase | | 3 | | 3 | | 3 | | 3 | | 3 | | 3 | | 3 | | 3 | | 3 | | 3 | | |
| Cytochrome b | | 1 | | 1 | | 1 | | 1 | | 1 | | 1 | | 1 | | 1 | | 1 | | 1 | | |
| Number of tRNAs | | 13 | | 13 | | 13 | | 13 | | 13 | | 13 | | 13 | | 13 | | 13 | | 15 | | |
| Number of rRNAs | | 2 | | 2 | | 2 | | 2 | | 2 | | 2 | | 2 | | 2 | | 2 | | 2 | | |

**Supplementary Table 2. Comparative characteristics of ten *B. mandrillaris* strains*.*** The mitochondrial genome size, number of protein-coding genes, tRNAs, and rRNAs of ten *B. mandrillaris* strains are summarized in a table.

**
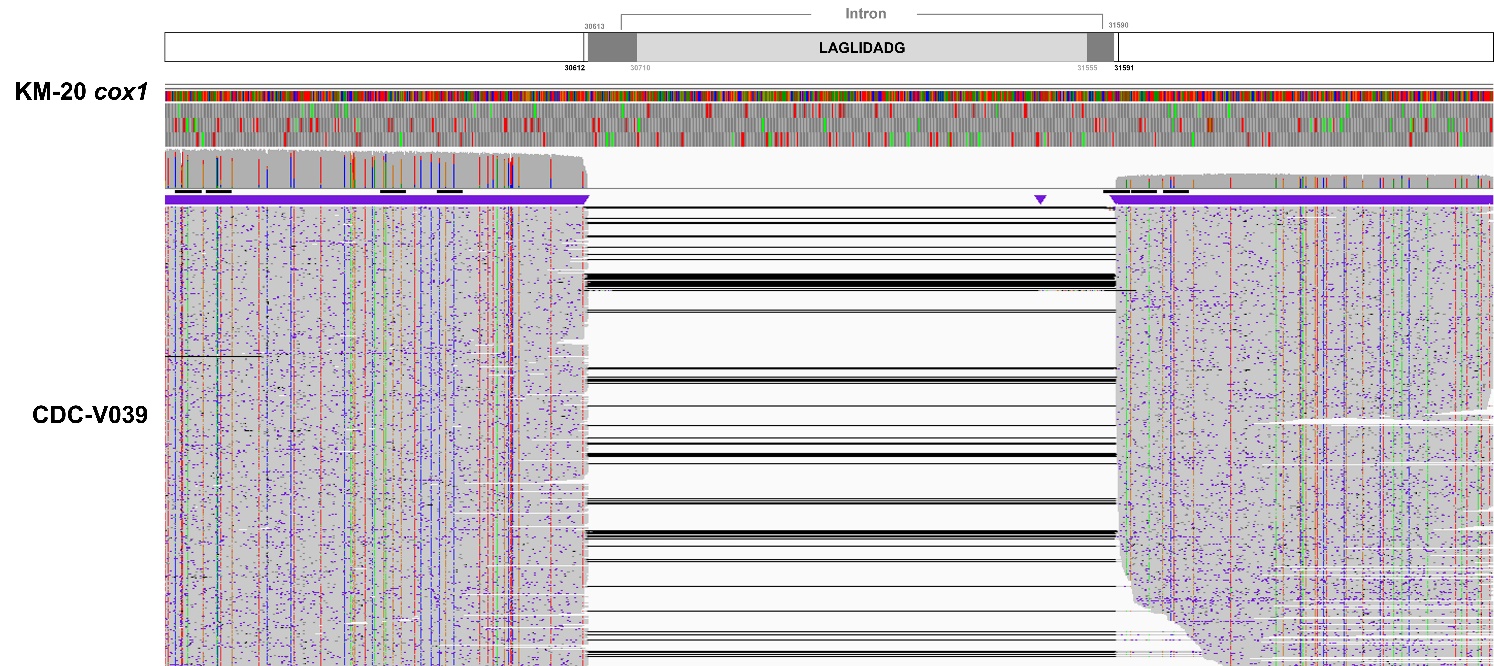
**

**Supplementary Fig. S2.** *Cox1* gene of *B. mandrillaris* KM-20 was interrupted by a LAGLIDADG-containing endonuclease. Five strains of *B. mandrillaris* have a LAGLIDADG endonuclease inserted in the *cox1* gene, namely strain KM-20, 2046, RP5, SAM, and OK1. Strain V039 and CDC-V039 do not contain any LAGLIDADG-endonuclease inserted in genes, whereas the remaining strains have a LAGLIDADG- endonuclease interrupting the 23S rRNA. The mapping result shows that the raw reads of strain CDC-V039 cannot span through the LAGLIDADG-containing intron in KM-20 *cox1* gene.

*-
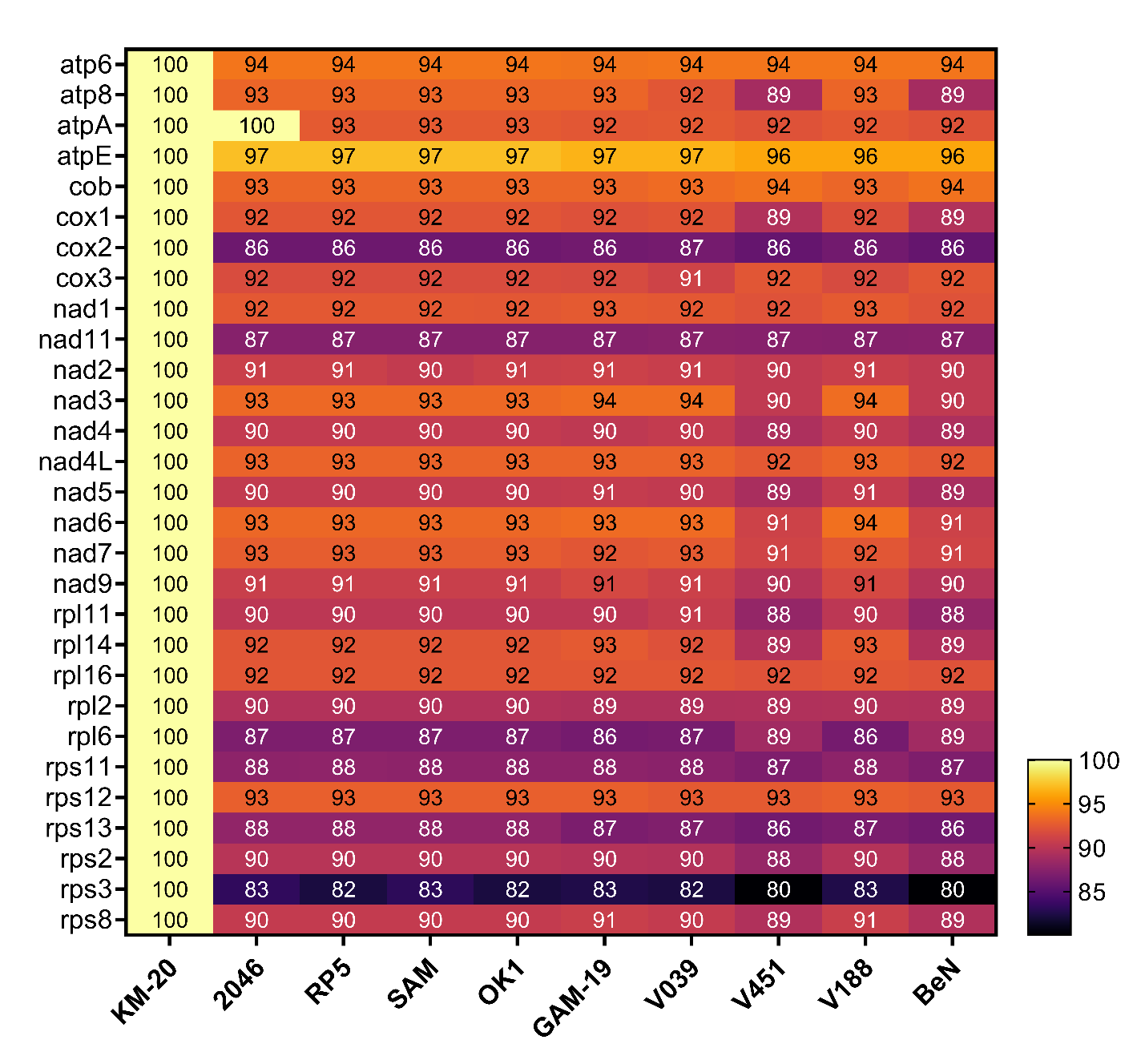
*

**Supplementary Fig. S3.** Heatmap representing the percentage identities of the mitochondrial coding-gene sequences across strains. The heatmap was derived from the percentage identity matrix calculated by Clustal Omega (v1.2.4). Among all mitochondrial genes, the *rps3* gene has the lowest percentage identity across strains.


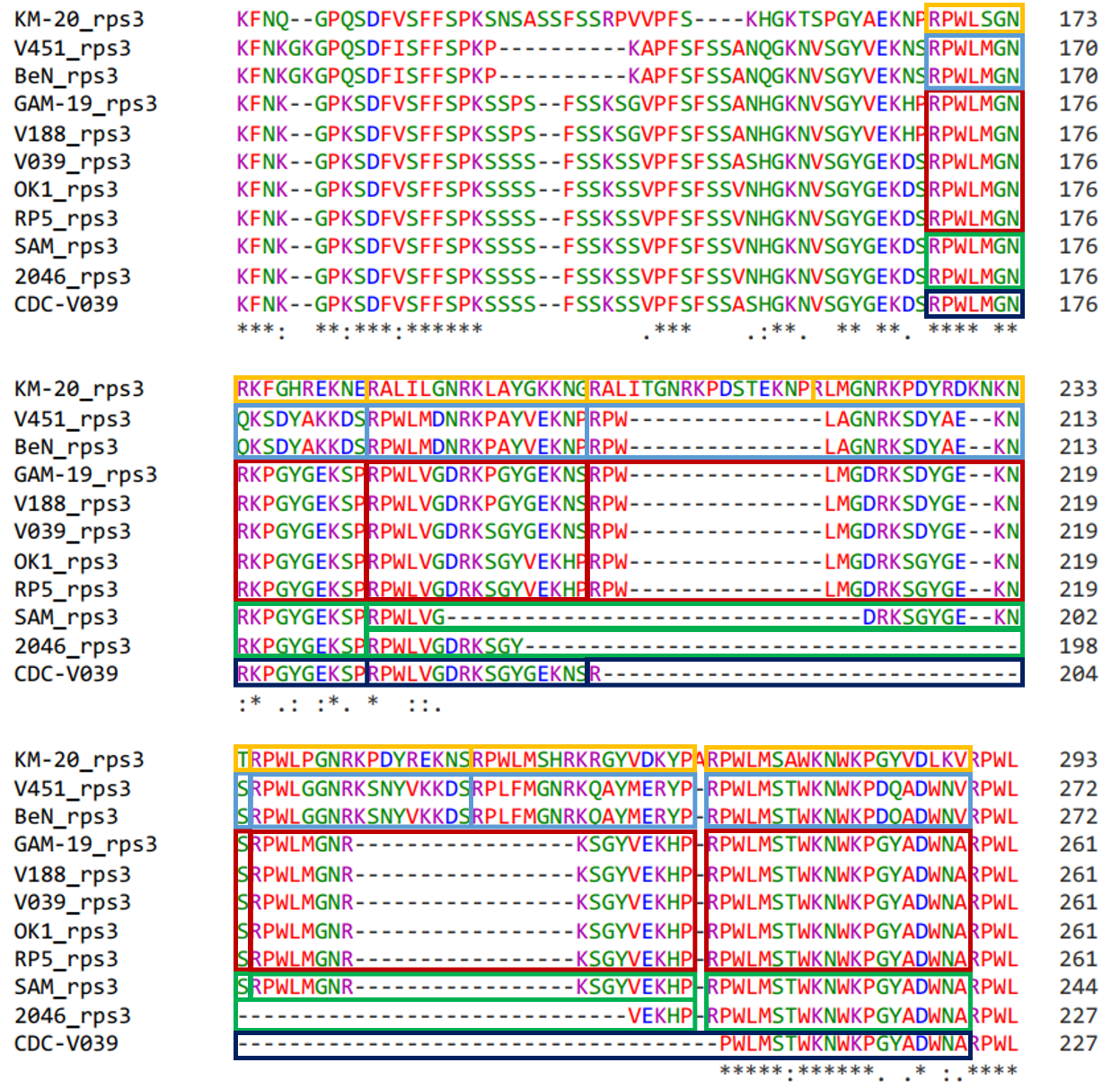


**Supplementary Fig. S4. CLUSTAL Multiple Sequence Alignment of rps3 from all *B. mandrillaris* strains.** The variation in rps3 arises from an array of protein tandem repeats. The repeating units are boxed, and each unit starts with Arginine (R), Proline (P), Tryptophan (W), and Leucine (L). The number of repeating units characterizes each strain: KM-20 has seven R units, which is the highest among all strains; V451 and BeN have six; GAM-19, V188, V039, RP5, and OK1 have five; SAM and 2046 have four and three R units respectively.


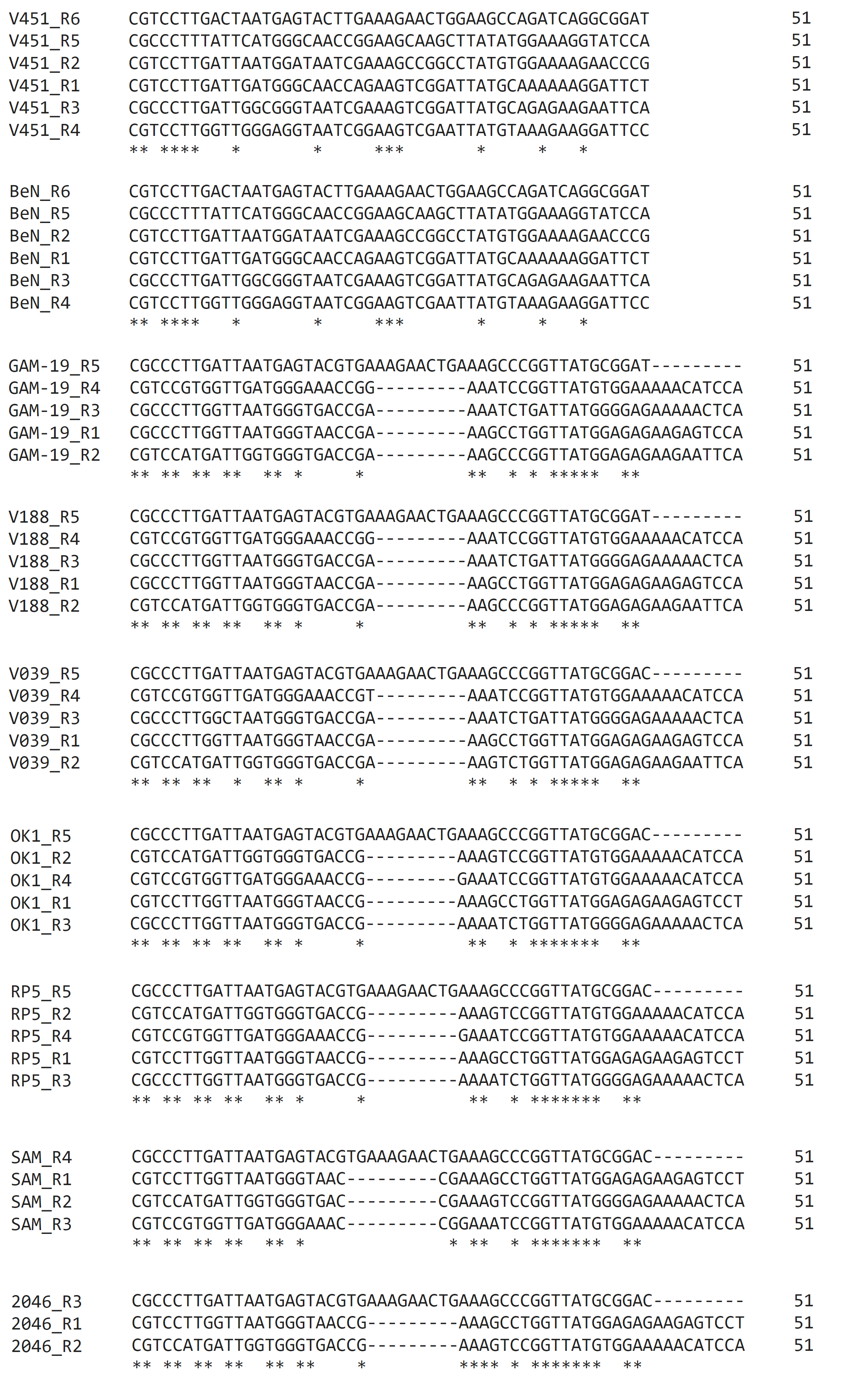


**Supplementary Fig. S5.** **Multiple sequence alignment of the nucleotide sequences within each repeat in *rps3* tandem repeat region among *B. mandrillaris* strains.** The nucleotide sequences are highly degenerated and can be differentiated from assembly algorithms such that the identified repeats are not due to assembly error and sequence collapse.


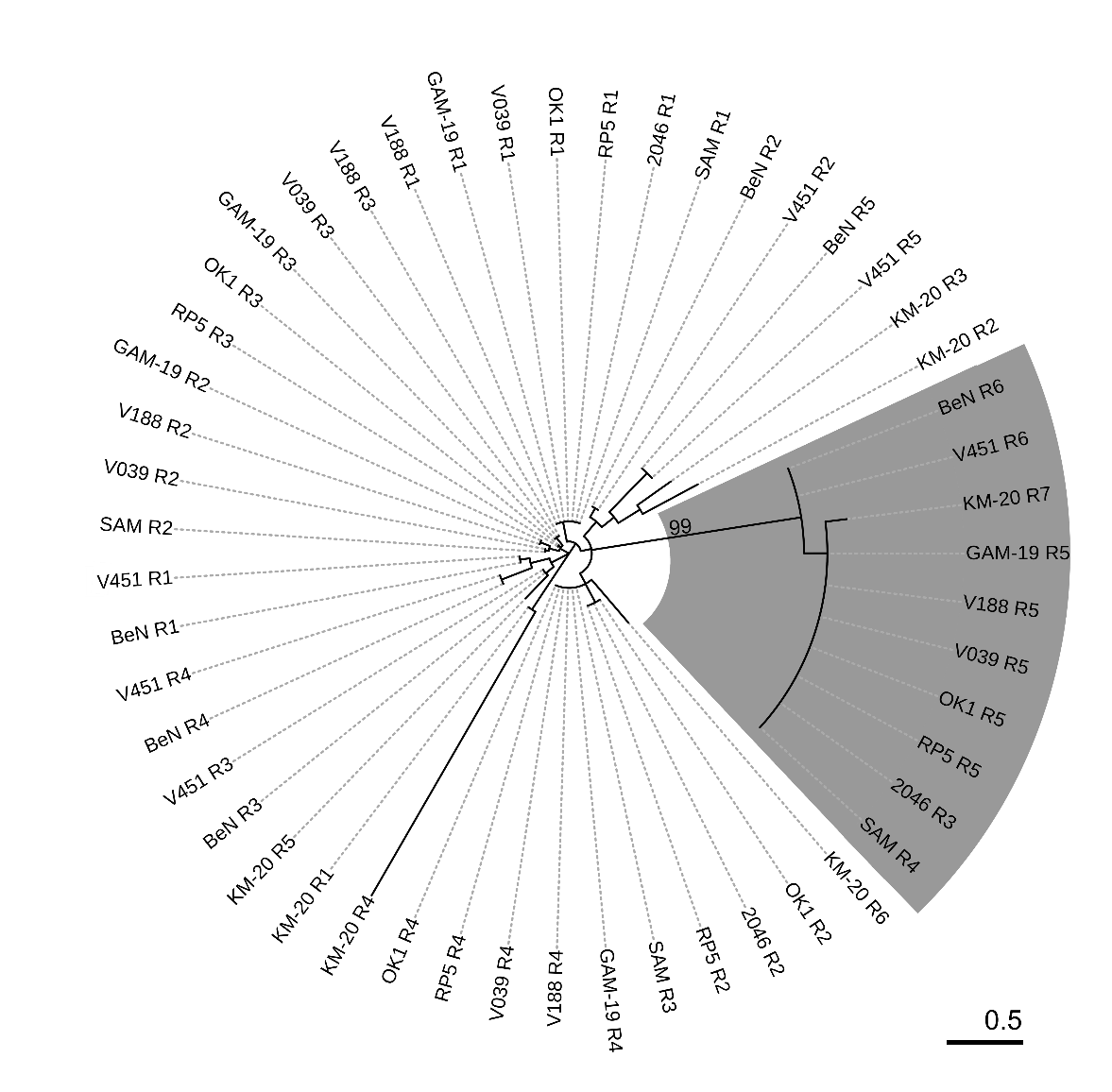


**Supplementary Fig. S6. Phylogenetic parent tree of *rps3* repeating units.** All repeating units of ten *B. mandrillaris* strains are phylogenetically analyzed. The repeating units nearest to the 3’-end of all strains clustered together to form a highly conserved branch with 99% bootstrap value and are colored in gray.


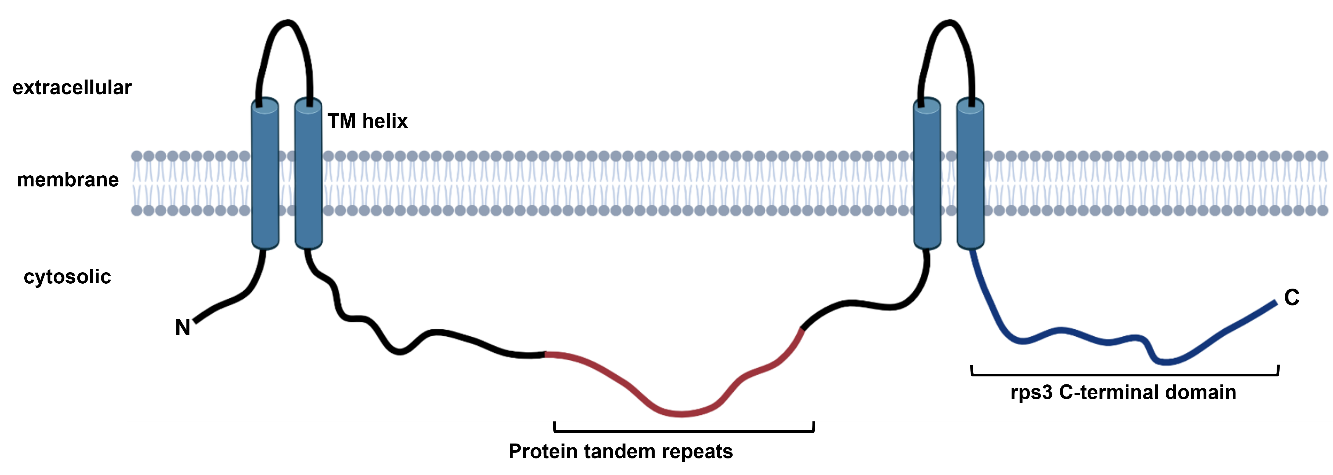


**Supplementary Fig. S7. Predicted structure of *B. mandrillaris* rps3.** The structure of rps3 was predicted to have four transmembrane helixes by InterPro88.0. The protein tandem repeat segment in rps3 was colored in red. Created with BioRender (https://biorender.com/).

**
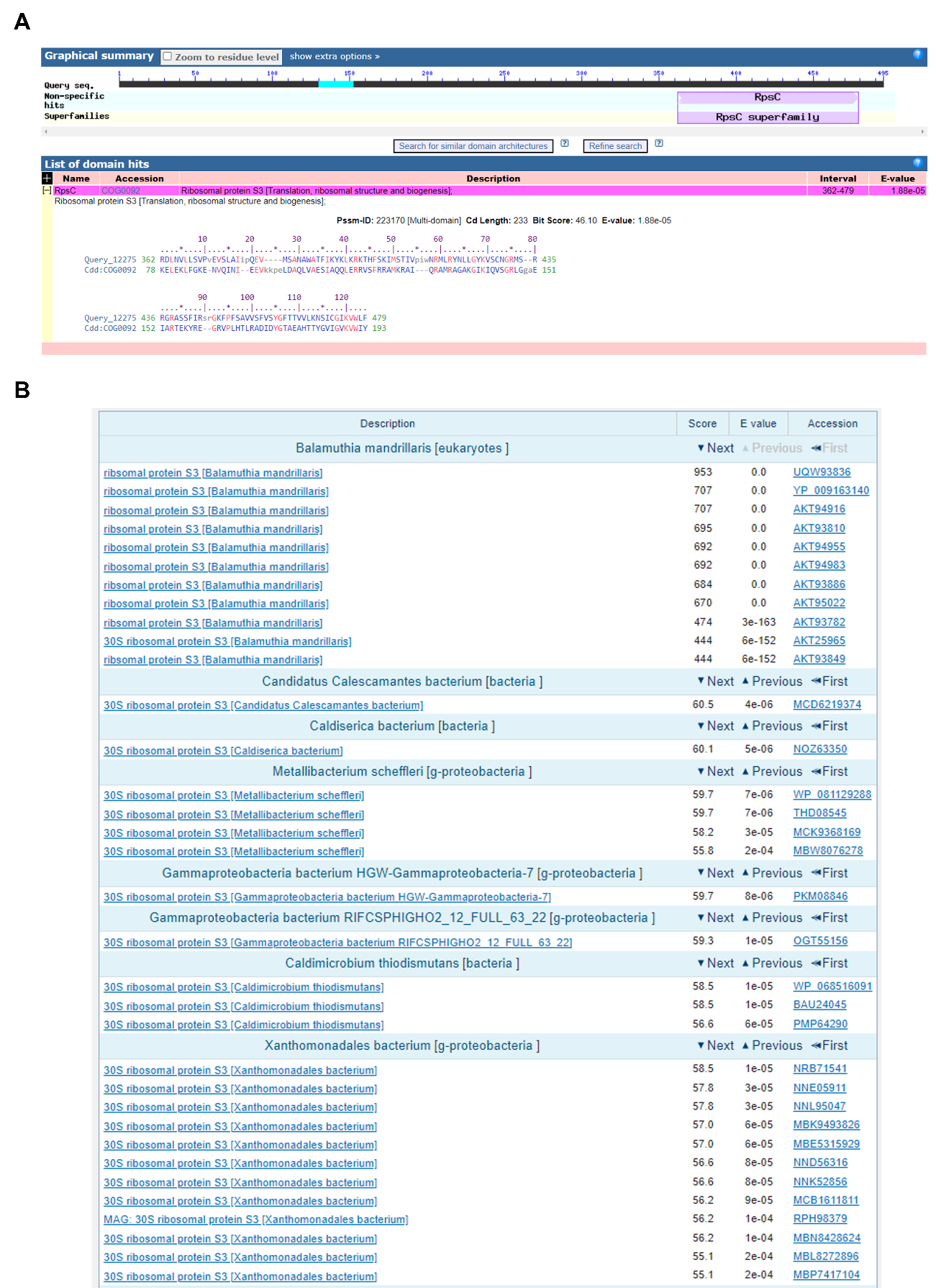
**

**Supplementary Fig. S8. BLASTp result of KM-20 rps3 protein sequence. (A)** The rps3 protein sequence of KM-20 was searched in BLASTp and the result was significantly matched to rps3 C-terminal domain with an e-value of 1.88e-05. **(B)** The top 100 BLASTp results of KM-20 rps3 are bacteria. Only the top eight search results were shown due to limited space.


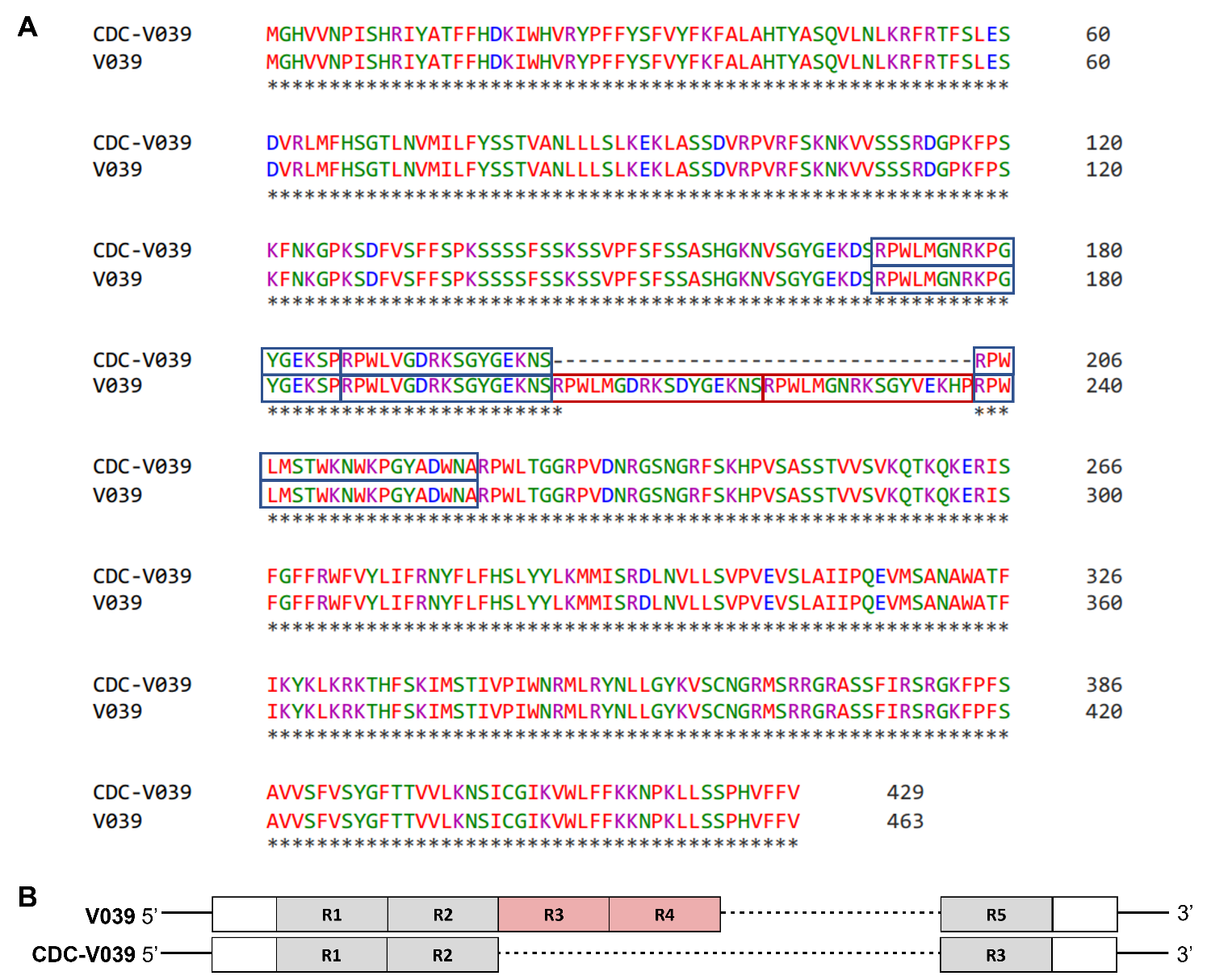


**Supplementary Fig. S9. Different numbers of repeating units exist in the same strain. (A)** Multiple sequence alignment of the rps3 protein sequences of V039 and CDC-V039. V039 has two extra repeating units than CDC-V039 and the extra units are boxed in red. V039 has five repeating units and CDC-V039 has three, the rest of the repeating units are boxed in blue and the amino acid sequences are identical. **(B)** The distribution of protein tandem repeats in rps3 inV039 and CDC-V039. The extra repeating units of V039 are colored in red.


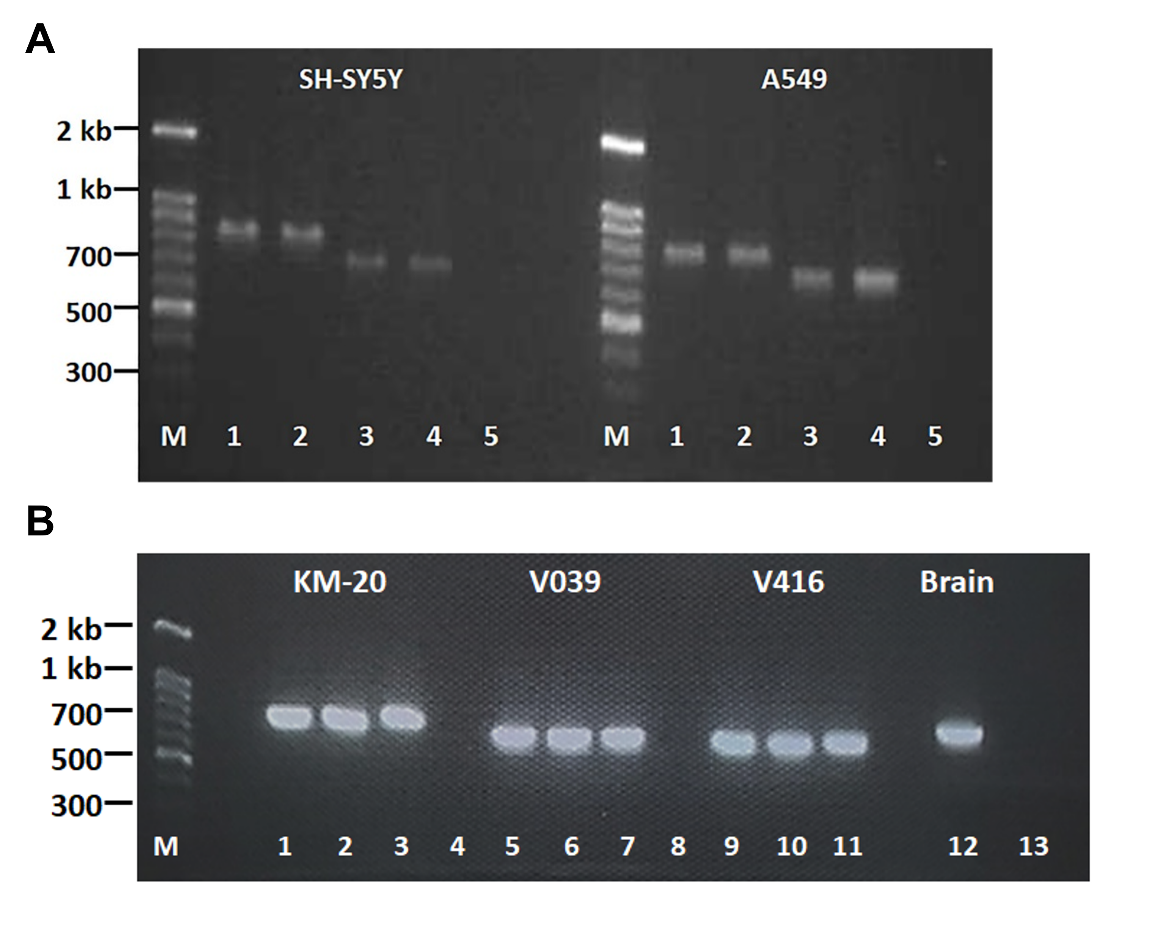


**Supplementary Fig. S10. *Rps3* gene expression in three different *B. mandrillaris* strains under different culture conditions.** **(A)** PCR amplification of the *B. mandrillaris rps3* gene in SH-SY5Y and A549 cell lines. Left: Lane 1, brain tissue from the case; Lane 2, KM-20 in A549; Lane 3, V039 in A549; Lane 4, V416 in A549; Lane 5, H_2_O. Right: Lane 1, brain tissue from the case; Lane 2, KM-20 in SH-SY5Y; Lane 3, V039 in SH-SY5Y; Lane 4, V416 in SH-SY5Y; Lane 5, negative control. **(B)** PCR amplification of the *B. mandrillaris rps3* gene at 25^◦^C, 37^◦^C, and 42^◦^C. Lane 1, KM-20 at 25^◦^C; Lane 2, KM-20 at 37^◦^C; Lane 3, KM-20 at 42^◦^C; Lane 4, H_2_O, Lane 5, V039 at 25^◦^C; Lane 6, V039 at 37^◦^C; Lane 7, V039 at 42^◦^C; Lane 8, H_2_O; Lane 9, V416 at 25^◦^C; Lane 10, V416 at 37^◦^C; Lane 11, V416 at 42^◦^C; Lane 12, brain tissue from the case; Lane 13, H_2_O.

**References**

Booton, G. C., Carmichael, J. R., Visvesvara, G. S., Byers, T. J., and Fuerst, P. A. (2003). Identification of *Balamuthia mandrillaris* by PCR assay using the mitochondrial 16S rRNA Gene as a target. *J. Clin. Microbiol.* 41, 453–455. doi: 10.1128/JCM.41.1.453-455.2003

Krasaelap, A., Prechawit, S., Chansaenroj, J., Punyahotra, P., Puthanakit, T., Chomtho, K., et al. (2013). Fatal Balamuthia amebic encephalitis in a healthy child: a case report with review of survival cases. *Korean J. Parasitol.* 51, 335–341. doi: 10.3347/kjp.2013.51.3.335

Intalapaporn, P., Suankratay, C., Shuangshoti, S., Phantumchinda, K., Keelawat, S., and Wilde, H. (2004). *Balamuthia Mandrillaris* meningoencephalitis: the first case in Southeast Asia. *Am. J. Trop. Med. Hyg.* 70, 666–669. doi: 10.4269/ajtmh.2004.70.666

Pélandakis, M., Serre, S., and Pernin, P. (2000). Analysis of the 5.8S rRNA gene and the internal transcribed spacers in Naegleria spp. and in *N. fowleri*. *J. Eukaryot. Microbiol.* 47, 116–121. doi: 10.1111/j.1550-7408.2000.tb00020.x

Schroeder, J. M., Booton, G. C., Hay, J., Niszl, I. A., Seal, D. V., Markus, M. B., et al. (2001). Use of subgenic 18S ribosomal DNA PCR and sequencing for genus and genotype identification of Acanthamoebae from humans with keratitis and from sewage sludge. *J. Clin. Microbiol.* 39, 1903–1911. doi: 10.1128/JCM.39.5.1903-1911.2001
